# Losing your edge: climate change and the conservation value of range‐edge populations

**DOI:** 10.1002/ece3.1645

**Published:** 2015-09-14

**Authors:** Evan M. Rehm, Paulo Olivas, James Stroud, Kenneth J. Feeley

**Affiliations:** ^1^ International Center for Tropical BotanyDepartment of Biological SciencesFlorida International UniversityOE 271,11200 SW 8th StreetMiamiFlorida33199; ^2^ The Fairchild Tropical Botanic GardenCoral GablesFlorida

**Keywords:** Ecotone, habitat loss, local adaptation, marginal populations, peripheral populations, range contraction, range expansion, species migrations

## Abstract

Populations occurring at species' range edges can be locally adapted to unique environmental conditions. From a species' perspective, range‐edge environments generally have higher severity and frequency of extreme climatic events relative to the range core. Under future climates, extreme climatic events are predicted to become increasingly important in defining species' distributions. Therefore, range‐edge genotypes that are better adapted to extreme climates relative to core populations may be essential to species' persistence during periods of rapid climate change. We use relatively simple conceptual models to highlight the importance of locally adapted range‐edge populations (leading and trailing edges) for determining the ability of species to persist under future climates. Using trees as an example, we show how locally adapted populations at species' range edges may expand under future climate change and become more common relative to range‐core populations. We also highlight how large‐scale habitat destruction occurring in some geographic areas where many species range edge converge, such as biome boundaries and ecotones (e.g., the arc of deforestation along the rainforest‐cerrado ecotone in the southern Amazonia), can have major implications for global biodiversity. As climate changes, range‐edge populations will play key roles in helping species to maintain or expand their geographic distributions. The loss of these locally adapted range‐edge populations through anthropogenic disturbance is therefore hypothesized to reduce the ability of species to persist in the face of rapid future climate change.

## Introduction

Anthropogenic climate change represents a major threat to global biodiversity (Urban 2015) and is causing strong ecological responses in many taxonomic groups across broad geographic areas (Parmesan 2006). Species can avoid climate‐driven extinctions through three principle mechanisms: (1) individuals of a species can acclimate to changes in climate; (2) species can adapt to changing climatic conditions in situ; and/or (3) species can shift their ranges to follow the geographic displacement of suitable climate conditions (Feeley et al. 2012). For many taxa, acclimation seems unlikely given that the rapid pace and large‐magnitude of current climate change will quickly push climatic conditions beyond tolerable limits (Jump and Peñuelas 2005). The remaining two options, species migration and adaptation, are not mutually exclusive and have occurred simultaneously in many species during past episodes of climate change (Davis and Shaw 2001). However, given the inability of many species to evolve at the extremely rapid rates required to keep pace with contemporary climate change (Quintero and Wiens 2013), the future of many species, and especially long‐lived species such as trees, will depend largely on their ability to shift their geographic ranges (Gienapp et al. 2008; Visser 2008; Chen et al. 2011; Hanski 2012 but see Skelly et al. 2007; Hoffmann and Sgro 2011). Indeed, many species are already responding to climate change through geographic range shifts (Parmesan 2006; Chen et al. 2011).

By their very nature, species range shifts take place at range edges, and therefore, range‐edge populations play a critical role during climate‐driven range shifts. At the leading range edge (i.e., the expanding or colonizing edge), populations can act as important stepping stones by serving as dispersal‐foci or by maintaining unique genetic adaptations that promote colonization of newly suitable areas (Gibson et al. 2009; Hannah et al. 2014). At the trailing range edge (i.e., the contracting or retreating edge), populations often exhibit high degrees of local adaptation and contain unique genotypes that may be necessary to species' persistence under future climates (Hampe and Petit 2005).

Biome boundaries, or ecotones, are defined by the convergence of many species' range edges and therefore represent geographic areas where locally‐adapted range‐edge populations are concentrated. Historically, many ecotones, such as those separating forests and savannahs or tundra, are also subject to high levels of human disturbance (Payette et al. 2001; Soares‐Filho et al. 2006; Körner 2012). For example, the highest rates of deforestation in the Amazon occur along the so‐called arc of deforestation at the frontier between the lowland forest and savannah/cerrado biomes, which by definition corresponds to the southerly range edge of many rainforest species (Soares‐Filho et al. 2006). Similarly, alpine treelines representing the upper range edge of many montane species have been subject to human disturbance for millennia (Körner 2012). Such geographically concentrated disturbances at ecotones may disproportionately remove range‐edge populations, impacting the overall ability of species' to persist during times of rapid climate change, and suggesting that these areas should be given greater prioritization as conservation targets.

The conservation value of range‐edge populations over evolutionary time scales has been well documented (Lesica and Allendorf 1995; Hampe and Petit 2005; Kawecki 2008). Over shorter time scales, the role of range‐edge populations for determining species' responses to rapid anthropogenic climate change remains largely underappreciated (but see Gibson et al. 2009). Range‐edge populations may become even more valuable under future climates as growing human populations will almost certainly increase the rate of large‐scale land‐use changes (Soares‐Filho et al. 2006) and accelerate climate change velocity (IPCC 2013). Despite their potential importance, recent efforts highlight that conservation networks often fail to adequately protect genetically unique range‐edge populations (Lefévre et al. 2013; Schueler et al. 2014). It is clearly critical that we develop a more comprehensive understanding and appreciation of the synergies between disturbance at species' range edges and the ability of species to respond to climate change.

Here, we use conceptual models to illustrate the potential for the locally‐adaptive traits contained in range‐edge populations to confer relative fitness advantages and dictate the short‐term responses of species to novel climates. In developing our conceptual models, we focus on tree species as an exemplar group because of the foundational roles that these long‐lived species play in many ecosystems. The theoretical background and empirical evidence of local adaptations in range‐edge populations has been summarized elsewhere (Bridle and Vines 2007; Eckert et al. 2008; Budd and Pandolfi 2010). As such, our assumption is that local adaptations that may be beneficial under future climates are already present in wild populations and that these adaptations can be quite common, especially in trees (Savolainen et al. 2007; Aitken et al. 2008; Leimu and Fischer 2008; Hereford 2009; Alberto et al. 2013).

We use two examples to illustrate how changes to climate coupled with habitat disturbance concentrated at areas where many species' range edges converge may be eliminating locally‐adapted populations that can be important for species' persistence under future climates. First, we discuss the cold range edges of tree distributions and the relationships between cold temperature events and future species distributions. In this example, we specifically discuss latitudinal and altitudinal treelines as they are distinct bioclimatic ecotones believed to be controlled by low temperature limitations on tree growth. In the second example, we focus on precipitation as the major determinant of species' distributions across lowland Amazonia as drought stress is known to greatly effect lowland tropical forest dynamics (Lewis et al. 2011). Given our heavy focus on tree species, caution should be taken when applying these conceptual models to other systems or taxonomic groups.

## When will Range‐edge Populations be Important?

**Figure 1:**
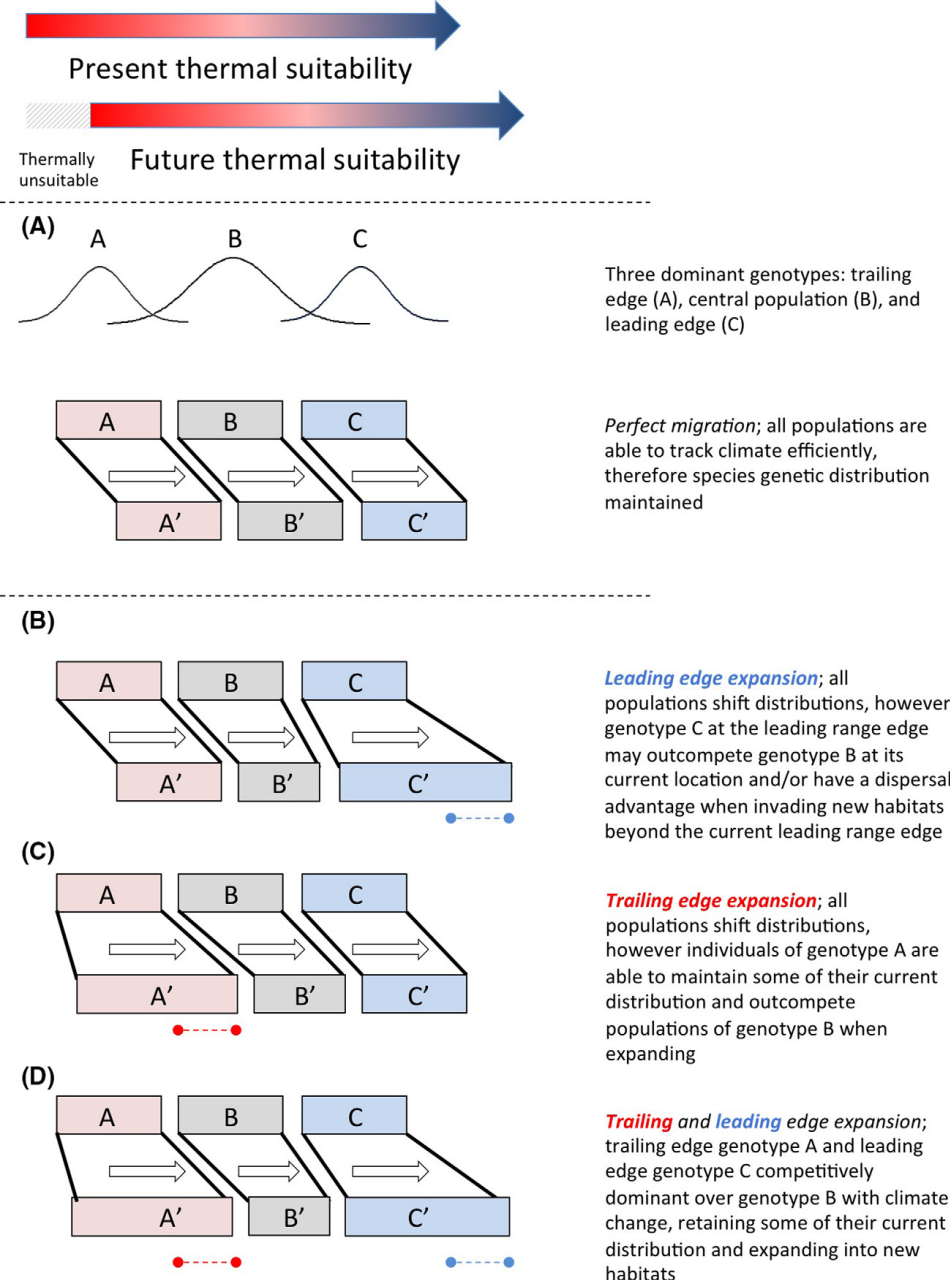
Conceptual models of distributional range shifts of locally‐adapted range‐edge populations during climate change.

Many predictions of how the geographic ranges of species will shift as a result of climate change are based on models that incorporate only a single summary climate variable such as mean annual temperature or total annual precipitation (TAP; Zimmermann et al. 2009). Under these predictions, a best‐case scenario is one in which species do not suffer from any sort of dispersal limitation and local populations track changing climates perfectly through time and space (Fig. 1A). However, such a scenario is clearly over‐simplified and unrealistic, as supported by the observation that the distributional shifts of many species' lag well behind concurrent climate shifts (Chen et al. 2011). One explanation for the observed lag in species' distributional shifts is that many species do not respond to just one measure of climate, but rather to multiple facets of climate including climatic extremes and variability (Zimmermann et al. 2009). Furthermore, within species, local populations may respond differently to changes in climate depending on the degree of local adaptation and the characteristics of the local environment. Understanding how species and local populations respond to various aspects of climate change (e.g., changes in means vs. variation of climate variables) will greatly improve our ability to predict species' persistence under climate change.

At the individual species level, when equilibrium climatic conditions are experienced, climate is commonly most suitable toward the center of the species' range and less suitable, or harsher, toward the range edges (Brown 1984). Range‐edge populations are often portrayed as being at their physiological limits because they are thought to be located at the edges of a species' climatic niche (MacArthur 1972) even though harsh climate at one species' range edge may be considered benign by another species. From the species' perspective, range‐edge climates are considered harsher than climate at the species' core as a result of an increased frequency and severity of extreme climate events, such as droughts, heat waves, or freezing episodes (Hoffman and Parsons 1997; Gaston 2003). These extreme climate events can play a strong role in determining the demographics and fitness of individuals and local populations (Gutschick and BassiriRad 2003; Jentsch et al. 2007; Lynch et al. 2014). There is also strong evidence that populations distributed across climatic gradients commonly show high levels of local adaptation, especially in tree species (Savolainen et al. 2007; Aitken et al. 2008; Leimu and Fischer 2008; Hereford 2009; Alberto et al. 2013). Localized range‐edge populations are therefore likely to maintain specialized genotypes that are particularly well‐adapted to the harsh or extreme climates that they experience.

If extreme climate events become more common as is often predicted (Kodra et al. 2011; Dai 2013; Trenberth et al. 2014), then range‐edge populations may have a fitness advantage (e.g., higher rates of survival) relative to the more central conspecific populations that are less tolerant of these events (Gutschick and BassiriRad 2003). According to this hypothesis, locally‐adapted range‐edge populations at both the leading and trailing range edges are predicted to expand their distributions and become increasingly abundant throughout species' ranges in the future (Fig. 1B–D). However, some geographic areas where many species' range‐edges converge (e.g., ecotones) may also be disproportionally affected by anthropogenic habitat disturbances relative to core populations. Therefore, range‐edge populations and their unique genotypes could be lost, reducing the ability of a species to rapidly colonize new climatically suitable habitats.

## The Leading Range Edges of Tree Species

While mean temperatures are increasing almost everywhere on Earth, the magnitude and frequency of extreme cold events are generally changing at much slower and idiosyncratic rates (Kodra et al. 2011). Therefore, locally‐adapted populations at species' cold range edges could become a key component for species survival. Cold adaptation in trees is not yet fully understood, but it is likely to be controlled by the complex interactions of dozens of different genes, each with small individual effect sizes (Howe et al. 2003). Local cold adaptations lead to the optimal balance between growth and avoidance of potentially damaging freezing events during the local growing season (Howe et al. 2003). Common‐garden experiments highlight that reductions in performance outside of the home site are usually attributed to asynchrony between an individual's genetically determined growth phenology and the optimal growing season at the planting site (Howe et al. 2003; Savolainen et al. 2007).

Local adaptations, such as the timing of tissue growth to minimize freezing damage while maximizing growing season length (Kollas et al. 2014), may be necessary for individuals to persist in species' current cold range edges. Indeed, latitudinal and altitudinal treelines are thought to occur at the point where cold temperatures directly limit tree growth, and therefore, optimizing growth during the short growing season while avoiding potentially lethal freezing events is a necessary prerequisite for populations inhabiting the treeline ecotone. Under future climate scenarios, local adaptations such as those found in treeline populations will also be necessary for populations to experience range expansion via colonization of areas becoming newly suitable under warmer temperatures. The lack of cold‐adapted individuals will effectively reduce the species' fundamental niche breadth and hence reduce the geographic extent of suitable areas.

Increasing mean temperatures have advanced the spring growth patterns of many North American trees, such that they now initiate growth earlier in the spring making them more vulnerable to spring frost events (Gu et al. 2008; Augspurger 2013). This pattern of increased freezing damage due to accelerated spring phenology can be seen across large geographic areas, which encompass the entire ranges of many tree species (Gu et al. 2008). If the selective pressures of avoiding spring frost events remain constant or increase under future climates, then we can predict that the more conservative growth patterns of cold‐adapted populations (i.e., delayed onset of spring growth) such as those found at treeline ecotones will be favored. If this is the case, warm‐adapted populations could be prevented from expanding or shifting their ranges. In contrast, cold‐adapted populations may maintain their current distributions as well as expand into areas that become climatically suitable under climate change (Fig. 1B).

While spring phenology is largely temperature dependent, growth cessation and bud set in the fall is mostly governed by photoperiod (Böhlenius et al. 2006). As tree species migrate poleward, especially into unforested areas beyond current latitudinal treelines, adjustment to the new photoperiods as a cue for fall bud set will be critical for minimizing exposure to the damaging frost events that become more likely as days become shorter. Given that local photoperiod will not be affected by global warming and if fall frost events exert a strong selective pressure on local populations, then we can predict that populations that are locally‐adapted to an area's specific photoperiod patterns will be favored at that location despite rising temperatures. As a result, populations occurring at species' current latitudinal cold range edges may be able to persist longer than would be predicted based on changes in temperature alone. Likewise, these range‐edge populations will be the best‐adapted to the photoperiods occurring in any areas poleward of the current distribution that become newly suitable under climate change.

In addition to local adaptations to cold temperatures and photoperiod, range‐edge populations are simply the closest populations in geographic space to the areas that are likely to become newly climatically suitable in the future. Hence, leading range‐edge populations represent the most likely dispersal sources for the individuals that will recruit into and colonize these newly suitable areas. Furthermore, in some tree and invasive plant species, populations at the cold or leading range edge show traits that enhance the potential for long‐distance seed dispersal (Cwynar and MacDonald 1987; Moran and Alexander 2014). Given that many species' current distributional shifts are lagging well behind climate shifts (Chen et al. 2011), the need for increased dispersal ability will become even more important in the future.

Large‐scale anthropogenic habitat disturbances near species' cold range edges could fragment and eliminate important leading range‐edge populations. For example, expansive forest clearing to make way for pastoral or agricultural lands has already occurred at many alpine treelines that are the cold range edges of numerous montane tree species (Körner 2012). Similarly, a combination of fires and deforestation of temperate forests at and around latitudinal treelines continues at a high rate (Payette et al. 2001). Even once anthropogenic disturbances have ceased at these cold or leading species range edges, repopulation of the former range limit occurs slowly, if at all (Holtmeier and Broll 2005). Therefore, it is likely that the loss and fragmentation of high‐latitude and high‐elevation forests is removing well‐adapted local populations that will be pivotal in allowing species to persist in their former and current ranges and expand their ranges under future climates, even in the absence of continuing human disturbance.

## The Trailing Range Edge of the Amazon – A Worst Case Scenario?

In the lowland tropics, temperature gradients are shallower than at higher latitudes (Wright et al. 2009). This means that across expansive lowland tropical forests, such as the Amazon, temperature remains relatively constant over very large geographic areas. In contrast, precipitation patterns can change markedly over much shorter distances and are believed to be a large driver of diversity and composition patterns throughout the lowland tropics (Engelbrecht et al. 2007). In lowland Amazonia, there is a distinct moisture gradient, with the south and southeastern Amazon being drier than areas to the west and north along the base of the Andes (Malhi and Wright 2004). In addition to being drier, the southern Amazon experiences large seasonal fluctuations in rainfall with a distinct and extended dry season (Malhi and Wright 2004). Over the last several decades, dry season length has increased in much of the southern Amazon, further intensifying moisture seasonality in this region leading to declines in forest vegetation (Hilker et al. 2014). Moreover, seasonality is expected to increase across the Amazon, with dry seasons becoming more pronounced and extreme drought events occurring more regularly, even in areas that are not currently affected by water shortages (Malhi et al. 2009).

The 2005 and 2010 Amazon droughts had pervasive, long‐lasting negative effects on tree growth and survival throughout the Amazon (Phillips et al. 2009; Lewis et al. 2011; Saatchi et al. 2013). As drought frequency and severity increase under future climates, local adaptations to drought tolerance will be important in allowing tree species to sustain their distributions. Identifying where drought‐adapted populations currently exist will therefore increase our ability to predict how many Amazonian species will respond to future climate change.

### Where might drought‐adapted populations occur?

Our understanding of intraspecific variation in drought tolerances in tropical trees is limited (McDowell et al. 2008; Craven et al. 2013). That said, it is reasonable to assume that many or most widespread Amazonian species exhibit some degree of local adaptation to moisture conditions given the ubiquity of genetic clines across temperature and moisture gradients in temperate species (Savolainen et al. 2007; McDowell et al. 2008). To highlight the potential for Amazonian plant species to exhibit local adaptations to moisture stress, we mapped the total annual precipitation (TAP; mm yr‐1) TAP (mm) and maximum climatological water deficit (MCWD; mm) across the distributions of 2157 widespread and large‐ranged tree species (estimated range size ≥300,000 km^2^) within the Amazon. MCWD is an integrative measure of the accumulative water stress experienced by plants in an area over the course of a year and has previously been found to be a strong predictor of species distributions as well as the location of humid tropical forests and other tropical biomes (Malhi et al. 2009). For a full description of the methods, see Data S1.

**Figure 2:**
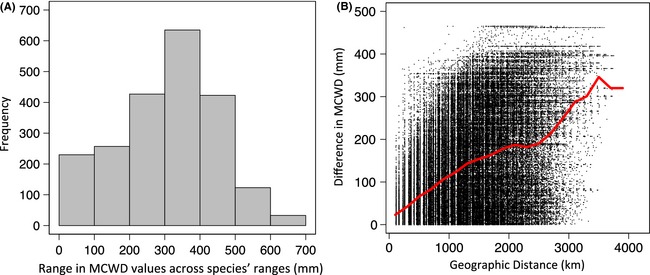
(A) Frequency distributions of the calculated range of MCWD (mm) across 2157 Amazon tree species' ranges with range sizes >300,000 km^2^. (B) Difference in MCWD and straight‐line geographic distance between all possible pairs of 1278 regularly spaced‐points (points located at the center of each degree latitude/longitude) within the lowland Amazon forest. The red line represents the running median of differences in MCWD within a moving window of 200 km distances.

The range of MCWD values occurring within the distributions of widespread tree species' was a median of 321 mm (95% confidence interval: 29.8–523 mm; Fig. 2A). Based on the geographic distribution of MCWD values in the Amazon, this corresponds to an estimated median range extent of more than 3000 km (Fig. 2B). Local adaptation to drought and summer moisture stress in populations of temperate species can be found over distances of just tens to hundreds of kilometers (Rehfeldt et al. 1999; St Clair et al. 2005). We found that Amazonian species experience large differences in MCWD across species' ranges, and that these differences occur over large geographic distances. Therefore, our findings support the contention that many of these tropical species are likely to maintain strong local adaptations to moisture stress conditions. More specifically, we predict that populations of widespread species that occur in the southern Amazon will be locally‐adapted to more extreme and/or prolonged drought conditions. If traits related to drought tolerance (e.g., water use efficiency) are limited to specific genotypes that are currently restricted to populations in the southern Amazon, then the individuals capable of persisting and populating the increasingly drier future Amazonian (Malhi et al. 2009) will likely originate from southern populations.

### Are we losing drought‐adapted populations?

**Figure 3:**
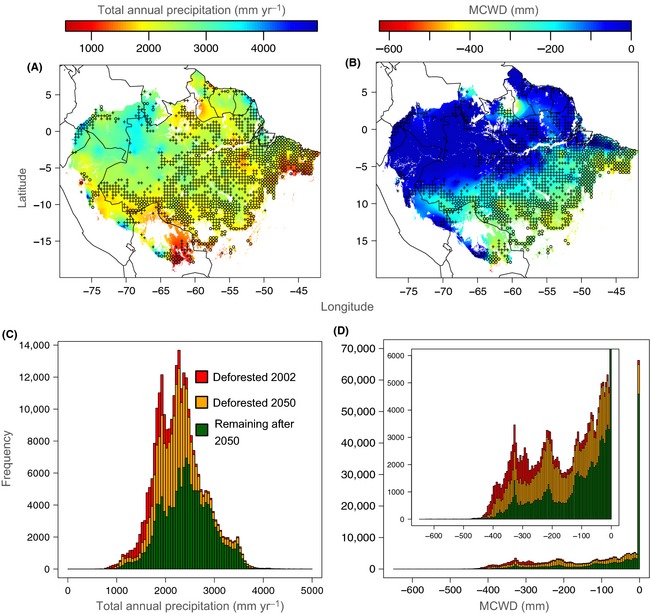
Current (A) total annual precipitation (TAP; mm/year) and (B) maximum climatological water deficit (MCWD; mm) across the Amazon mapped at a resolution of 2.5 arc min based on the WorldClim high‐resolution extrapolated climate database. Circles represent areas that have been mostly deforested as of 2002, and crosses represent areas predicted to be mostly deforested by 2050 under a BAU deforestation scenario (Soares‐Filho et al. 2006). Estimated (C) TAP and (D) MCWD frequency of areas deforested as of 2002, areas predicted to be deforested by 2050, and remaining as forest after 2050. Inset in (D) is an enlarged version of the larger histogram shown in (D).

The southern Amazon is widely known as “the arc of deforestation” due to large‐scale forest clearing for agricultural development (Soares‐Filho et al. 2006). Beyond simply reducing total forest area, this geographically clustered deforestation may be having the additional negative effect of eliminating the populations that are best‐adapted to the dry and seasonal conditions that will be increasingly widespread in the future. To illustrate how deforestation relates to local climate, we mapped current total annual precipitation and MCWD across the Amazon (resolution of 2.5 arc min based on the WorldClim high‐resolution extrapolated climate database; Hijmans et al. 2005). We then overlaid areas that were mapped as being deforested as of 2002 and in areas that are predicted to be deforested by 2050 under a spatially explicit model of deforestation assuming business‐as‐usual (BAU) rates and constraints (Soares‐Filho et al. 2006). These analyses clearly show that deforestation is currently occurring and is predicted to occur, predominantly in those areas that have lower annual precipitation (Fig. 3A, C) and higher seasonality of precipitation (Fig. 3B, D) such as the Amazon‐cerrado ecotone.

To help illustrate how deforestation in the southern Amazon could potentially hinder a species ability to adapt to future climate, we next mapped future climate analogs under the 2002 and 2050 BAU deforestation scenario (future precipitation and MCWD were based on six leading general circulation models at a resolution of 2.5 arc min with projections until 2070 under the RCP8.5 emissions scenario; Fig. S1). Using the future climate (TAP and MCWD) predictions, we then identified and tallied the areas within the lowland Amazon forest that have analogous current climates. We then calculated the relative reduction (% decrease) in the number of current climate analogs corresponding to each cell due to the loss of area under current (2002) and predicted future (2050 BAU) deforestation.

**Figure 4:**
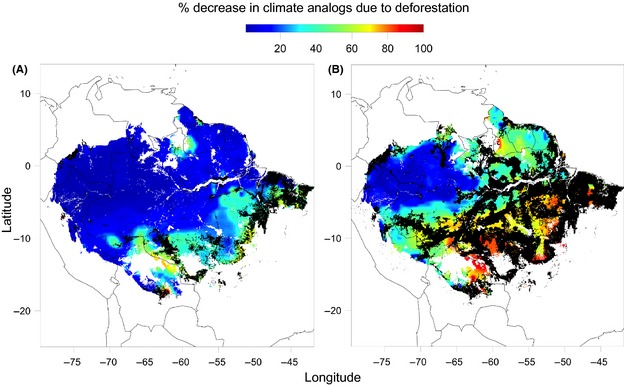
The median percent reduction in number of potential future climate analog source populations in the Amazon under 2070 climate projections accounting for climate analogs lost due to (A) deforestation as of 2002 and (B) deforestation for 2050 under a BAU scenario (deforested areas are mapped as black). Values represent the median estimates generated using climate projections from six individual general circulation models (GCMs): NCAR_CCSM4, GFDL_CM3, CSIRO_ACCESS1.0, MOHC_HADGEM2, IPSL_CM5A_LR, and MIROC_MIROC5, and the RCP8.5 emissions scenario. Black represents a complete loss (100% reduction) of available climate analogs based on losses from deforestation and the introduction of novel climates.

We found that current deforestation reduces the extent of climate analogs (i.e., the extent of populations that will be available to populate future Amazonian forests) by an average of 16% (Fig. 4A) and that future deforestation will reduce the extent of climate analogs by an additional 36% (Fig. 4B). These results highlight the fact that because deforestation is occurring predominantly in the dry seasonal forest of the southern Amazon, and because the Amazon as a whole is predicted to become increasingly dry and seasonal, the effects of deforestation will extend well beyond its footprint. In other words, deforestation is reducing the number of drought‐resistant populations that can potentially immigrate into the increasingly widespread dry conditions that are analogous to conditions that are currently restricted to the southern Amazon.

Our analyses are meant to highlight the potential importance of range edge populations in trees and hence the danger of losing these populations through deforestation. The actual effects of deforestation on species persistence under climate change will be highly species‐specific and will depend on many different factors including the degree of local adaptation, the overlap between the species' geographic ranges and deforestation, and the species' dispersal ability, including the ability to cross‐disturbed/modified habitats (Feeley and Rehm 2012; Urban 2015). Understanding what important genetic traits are lost during human disturbance at forest ecotones can be an important component of understanding forest regeneration and dynamics under future climates, especially as deforested areas are abandoned and allowed to regrow (Chazdon et al. 2009).

## Caveats, Applications, and Future Directions

During future climate change, extreme climatic events will become more frequent and severe across large geographic areas, significantly affecting the ability of many species to persist into the future (Zimmermann et al. 2009; Reyer et al. 2013). In addition, asynchronous rates of change in different climate variables will result in novel climates being introduced throughout the globe (Williams et al. 2007). Using relatively simple conceptual models and empirical analyses, we highlight how locally‐adapted populations occurring at tree species range edges are perhaps the best suited to survive extreme climate events and persist under future novel climates. We also discuss and show how large‐scale habitat disturbances are removing unique range‐edge populations, predominantly at ecotones, potentially hindering species' abilities to respond to climate change.

Our models and predictions have some notable caveats. We focused on just two main forested systems, treelines and the southeast Amazon, as these biome boundaries are exemplary of areas with high species range edge convergence coupled with large‐scale anthropogenic disturbance. Notably, our arguments concerning latitudinal and alpine treelines would be greatly improved by further detail concerning the rates and patterns of deforestation at these ecotones. Regardless, we chose these two systems to underline the main points of our models, which were designed to show that human disturbances often can and do remove important range edge populations. Species range edges can occur anywhere across globe and not only at major biogeographic boundaries. Therefore, the application of our conceptual models at the species level will depend on the local intensity of land‐use change and the removal of edge as opposed to more core populations, which will vary depending on the geographic region and species in question.

We also did not explicitly incorporate the phenotypic responses of local genotypes. We believe that this is justified due to the unprecedented magnitude and pace of current climate change which will quickly introduce novel environmental conditions that are likely to be beyond what species can tolerate through plastic responses and acclimation (Jump and Peñuelas 2005; Valladares et al. 2007). Plasticity itself is an evolvable trait, but adaptive plasticity is most likely to evolve when cues reliably predict environmental conditions (van Kleunen and Fischer 2005; Nicotra et al. 2010). Given the current pace of climate change and that environmental cues may become progressively unreliable and environmental conditions unpredictable, rapid evolutionary responses of plasticity and other traits are unlikely in organisms such as trees that are long‐lived and have long generation times (van Kleunen and Fischer 2005; Hanski 2012; Merilä 2012). Although rapid adaptation can occur over a surprisingly small number of generations, the long generation times in trees means that any adaptive responses to climate change will likely lag behind changes in climate (Jump and Peñuelas 2005; Quintero and Wiens 2013). As such, we limit our discussion to the beneficial traits currently expressed by locally‐adapted genotypes and we do not specifically address adaptive phenotypic plasticity or microevolutionary processes.

We give special attention to the responses of locally‐adapted range‐edge populations to only temperature and precipitation as these two variables are often found to be the most important abiotic determinants of range limits in trees (Cahill et al. 2014). Under future climate scenarios, it is likely that the relative importance of range‐edge populations to total species' persistence will increase dramatically. However, other abiotic factors (e.g., additional climatic factors, soil conditions) and biotic factors (e.g., herbivory rates, competition) will also likely influence species' responses to climate change. With rapid advancements in the breadth and complexity of predictive tools, we should begin to incorporate both climatic and nonclimatic factors into our conceptual models to create more realistic and accurate predictions of species shifts under future climates (Svenning et al. 2014).

Theoretical efforts toward predicting where and when local adaptations occur in wild populations have seen great advances over the past decades (Merilä 2012). However, empirical evidence often contradicts theory and shows large variations in where and when local adaptations occur (Sagarin et al. 2006; Bridle and Vines 2007). In order to understand and conserve important range edge populations, we must first identify the populations within species' ranges that hold genotypes that may be beneficial under future climate (e.g., see Fitzpatrick and Keller 2015). Future research into identification and genetic mapping of entire species' ranges has large implications for ecosystem function and persistence under future climates.

Specific focus should be given to identifying the traits within locally‐adapted populations that are predicted to become more important under future climate conditions (e.g., heat and drought tolerance). Identifying the responsible genetic controls of these traits will aid in conservation efforts and advance our ability to accurately forecast where and when locally‐adapted range‐edge populations will be important determinants of species persistence. Even in well‐studied traits, such as cold temperature adaptation in northern hemisphere trees, we still have a relatively basic understanding of how specific genes control phenotypic expression and how genes are linked or interact (Howe et al. 2003; Savolainen et al. 2007). Without knowing the constraints to heritable variation in traits under selection, we are unable to make accurate predictions about rapid evolutionary adaptation in populations.

The recent and rapid advances in molecular techniques may allow for the disentangling of genetic controls on beneficial traits and allow researchers to weigh the importance of range shifts versus rapid evolutionary adaptation in a timeframe relevant for contemporary climate change. Work of this nature should focus on intraspecific population genetics across a wide variety of taxa and functional groups that exhibit different life history strategies. Efforts such as these will greatly increase our ability to predict species' range shifts and extinctions and therefore help to most effectively focus the limited resources available for conservation.

## Conflict of Interest

None declared.

## Supporting information


**Data S1.** Methods.

**Figure S1.** The percent reduction in number of potential future climate analog source populations in Amazonia under 2070 climate projections accounting for climate analogs lost due to deforestation as of 2002 and under future BAU deforestation for 2050. Black represents a 100% reduction in available climate analogs based on losses from deforestation and the introduction of novel climates.
