# Supplementary material for: Losing your edge: climate change and the conservation value of range‐edge populations
Source: Ecol Evol. 2015 Sep 14;5(19):4315–26. doi: 10.1002/ece3.1645 (PMC4667833; doi:10.1002/ece3.1645)
Supplement: Supplementary file 1 — Data S1. Methods. [file ECE3-5-4315-s001.doc]

**Methods (871)**

**Precipitation and water stress across widespread species’ ranges**

For all analyses the lowland Amazon forest was defined as areas of Amazonia mapped as having or once having forest cover (i.e., forest or deforested areas) according to (Soares-Filho et al. 2006) and with an elevation of ≤ 500 meters above sea level. Within this area we mapped climate at a resolution of 2.5 arc minutes (corresponding to between 20.3 and 21.5 km^2^ in our study area) based on the WorldClim high-resolution extrapolated climate database (<http://www.worldclim.org/>; (Hijmans et al. 2005)). Specific climate variables included in the analyses were total annual precipitation (TAP; millimeters [mm]) and the Maximum Climatological Water Deficit (MCWD; mm). MCWD is an integrative measure of the accumulative water stress experienced by plants in an area over the course of a year(Malhi et al. 2009). MCWD is estimated as the most negative value of climatological water deficit (CWD) over a year, where the monthly change in CWD is equal to precipitation (P; mm/month) - evapotranspiration (E; mm/month) such that for month i: CWD_i_ = CWD_i-1_ + P_i_ - E_i_; Max(CWD_i_) = 0; CWD_0_ = CWD_12_; MCWD = Min(CWD_1_…CWD_12_). In calculating MCWD, we started the twelve-month calculation cycle at the wettest month of the year at which point the soil was assumed saturated (i.e., CWD = 0). We did not use direct estimates of E but instead used a fixed estimate of E = 100 mm/month which approximates the average evapotranspiration rate observed in humid tropical forests under non-drought conditions(Malhi et al. 2002; Fisher et al. 2009, 2011).

In order to look at the distribution of climatic variables within the geographic distributions of species, we used the Amazonian plant species range maps of (Feeley et al. 2012) downloaded through the Map of Life (<http://map.mol.org/>). Within the mapped ranges of 2157 plant species with range sizes estimated as ≥300,000 km^2^, we extracted all MCWD values and calculated the range of MCWD values across the species full range [Max(MCWD) - Min(MCWD)]. The distributional range area cutoff of 300,000 km^2^ is the approximate median range size of Amazonian tree species(Feeley & Silman 2009; ter Steege et al. 2013) and was used to distinguish widespread from small-ranged species. We excluded species with smaller range sizes since they may be habitat specialists, which by definition are less likely to have populations locally adapted to different hydrologic regimes. We assume that species with distributions spanning broader ranges of MCWD (i.e. higher values in Fig 2a) have a greater potential for local adaptations to moisture conditions.

To determine the relationship between differences in MCWD and geographic distances we extracted MCWD values at 1,278 regularly-spaced points (points located at the center of each degree latitude/longitude) within the lowland Amazon forest. For all possible pairs of points (203,841 unique point combinations) we then calculated the absolute difference in MCWD and the straight-line geographic distance, and finally estimated the running median of differences in MCWD within a moving window of 200 km distance.

**Deforestation and future climate analogs across the Amazon**

We used climate analogs as a proxy for the current and future distribution of locally adapted populations for a given species. We assumed locally adapted populations will track suitable climates as they shift in space due to climate change.

We began by mapping current TAP and MCWD values within the Amazon at a resolution of 2.5 arc minutes based on the WorldClim high-resolution extrapolated climate database (<http://www.worldclim.org/>; (Hijmans et al. 2005)). We then overlaid areas that were mapped as being deforested as of 2002 and in areas that are predicted to be deforested by 2050 under a spatially explicit model of deforestation assuming Business-As-Usual (BAU) rates and constraints(Soares-Filho et al. 2006).

To show how deforestation in the southern Amazon could hinder a species ability to adapt to future climate, we mapped future climate analogs under the 2002 and 2050 BAU deforestation scenario. Future monthly precipitation for the lowland Amazon was estimated based on six leading General Circulation Models (GCMs): NCAR_CCSM4, GFDL_CM3, CSIRO_ACCESS1.0, MOHC_HADGEM2, IPSL_CM5A_LR, and MIROC_MIROC5. Future projections were for the 2070s under the RCP8.5 emissions scenario. All models were downscaled to a resolution of 2.5 arc minute based on statistical downscaling downloaded from the International Centre for Tropical Agriculture (CIAT) and the CGIAR Research Program on Climate Change, Agriculture and Food Security (CCAFS; <http://www.ccafs-climate.org/>).

Using the projected monthly precipitation values, we calculated the future TAP and MCWD predicted for each grid cell under each of the individual GCMs. Using the future climate (TAP and MCWD) predictions, we then identified and tallied the areas within the lowland Amazon forest that have analogous current climates. Climate analogs were not allowed to occur outside the current boundaries of the Amazon forest as defined above. When identifying climate analogs, we allowed for a difference of ≤ ± 10% in both TAP and MCWD. We then calculated the relative reduction (% decrease) in the number of current climate analogs corresponding to each cell’s future climate due to the loss of area under current (2002) and predicted future (2050 BAU) deforestation. To generate an ensemble prediction, we calculated the median reduction in climate analogs available to each cell due to current and future deforestation across the six individual GCMs (individual model values can be found in Supp Fig1).

**References**

Feeley, K. J., and M. R. Silman. 2009. Extinction risks of Amazonian plant species. Proceedings of the National Academy of Sciences **106**:12382–12387.

Feeley, K., Y. Malhi, P. Zelazowski, and M. Silman. 2012. The relative importance of deforestation, precipitation change, and temperature sensitivity in determining the future distributions and diversity of Amazonian plant species. Global Change Biology **18**:2636–2647.

Fisher, J. B. et al. 2009. The land-atmosphere water flux in the tropics. Global Change Biology **15**:2694–2714.

Fisher, J. B., R. J. Whittaker, and Y. Malhi. 2011. ET come home: potential evapotranspiration in geographical ecology. Global Ecology and Biogeography **20**:1–18.

Hijmans, R. J., S. E. Cameron, J. L. Parra, P. G. Jones, and A. Jarvis. 2005. Very high resolution interpolated climate surfaces for global land areas. International Journal of Climatology **25**:1965–1978. John Wiley & Sons, Ltd.

Malhi, Y., L. E. O. C. Aragão, D. Galbraith, C. Huntingford, R. Fisher, P. Zelazowski, S. Sitch, C. McSweeney, and P. Meir. 2009. Exploring the likelihood and mechanism of a climate-change-induced dieback of the Amazon rainforest. Proceedings of the National Academy of Sciences **106**:20610–20615.

Malhi, Y., E. Pegoraro, A. Nobre, M. G. P. Pereira, J. Grace, A. Culf, and R. Clement. 2002. Energy and water dynamics of a central Amazonian rain forest. Journal of Geophysical Research **107**:8061.

Soares-Filho, B. S., D. C. Nepstad, L. M. Curran, G. C. Cerqueira, R. A. Garcia, C. A. Ramos, E. Voll, A. McDonald, P. Lefebvre, and P. Schlesinger. 2006. Modelling conservation in the Amazon basin. Nature **440**:520–523.

Ter Steege, H. et al. 2013. Hyperdominance in the Amazonian tree flora. Science **342**:6156.

Fig S1: The percent reduction in number of potential future climate analog source populations in Amazonia under 2070 climate projections accounting for climate analogs lost due to deforestation as of 2002 and under future BAU deforestation for 2050. Black represents a 100% reduction in available climate analogs based on losses from deforestation and the introduction of novel climates. Climate projections were based on 6 leading General Circulation Models (GCMs) and paired maps represent reduction in number of potential future climate analog source populations accounting for loss due to deforestation as of 2002 and 2050, respectively: NCAR_CCSM4 (a,b), GFDL_CM3 (c,d), CSIRO_ACCESS1.0 (e,f), MOHC_HADGEM2 (g,h), IPSL_CM5A_LR (i,j), and MIROC_MIROC5 (k,l), and the RCP8.5 emissions scenario.
