# Supplementary figures and images for: Losing your edge: climate change and the conservation value of range‐edge populations
Source: Ecol Evol. 2015 Sep 14;5(19):4315–26. doi: 10.1002/ece3.1645 (PMC4667833; doi:10.1002/ece3.1645)

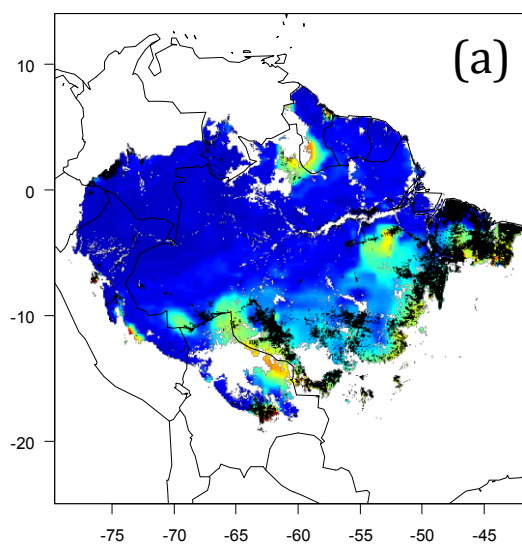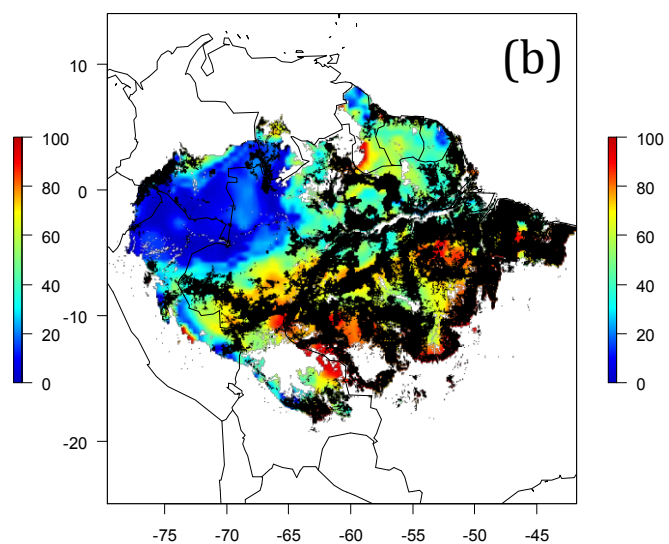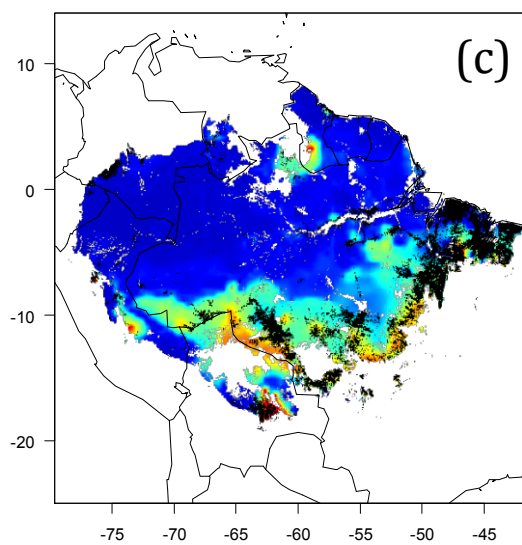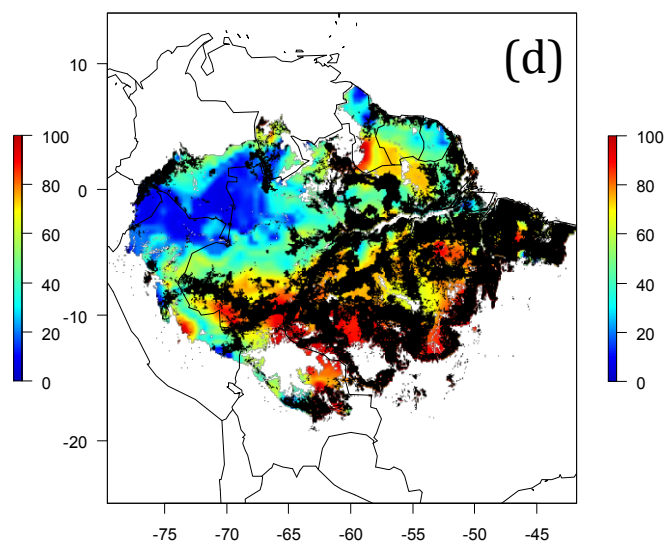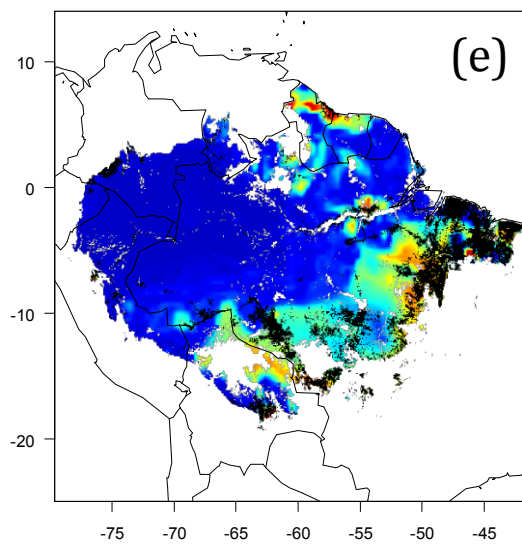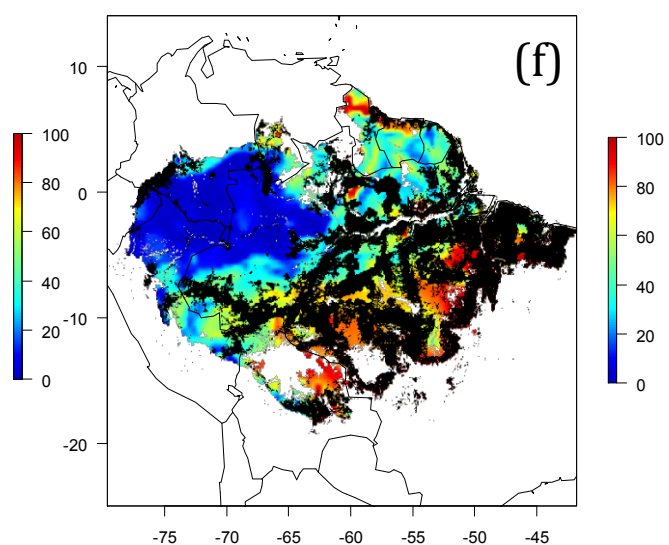

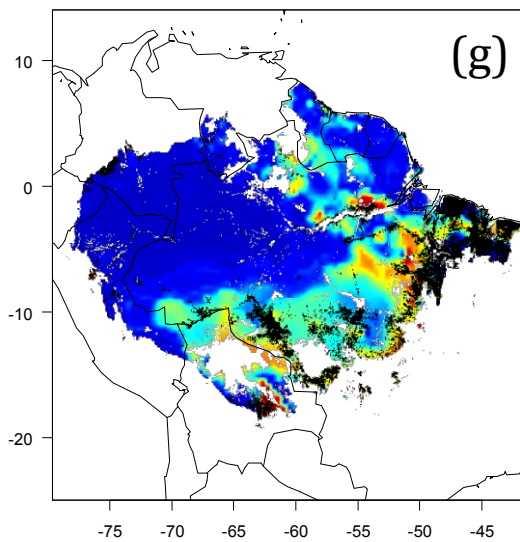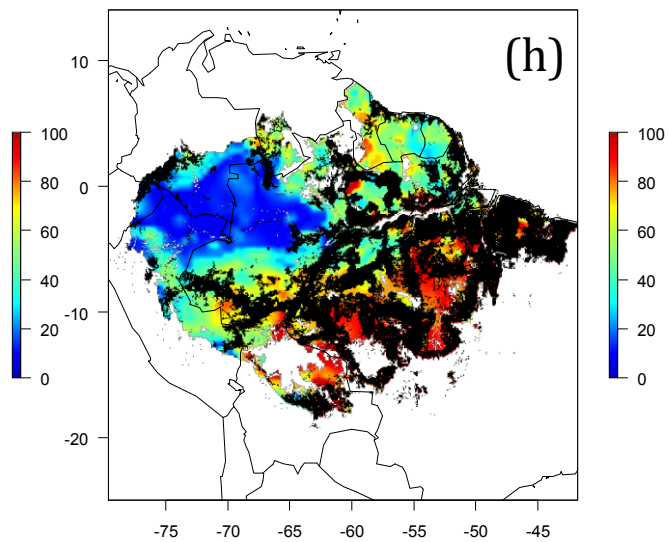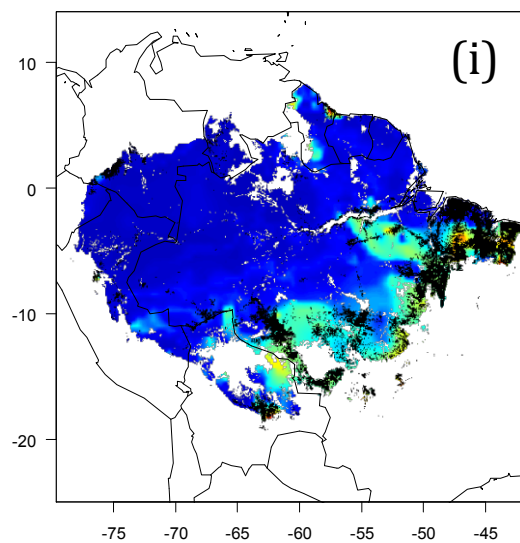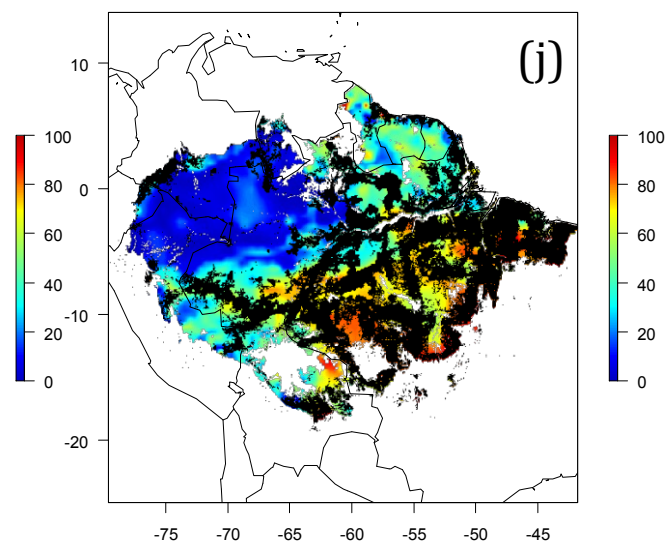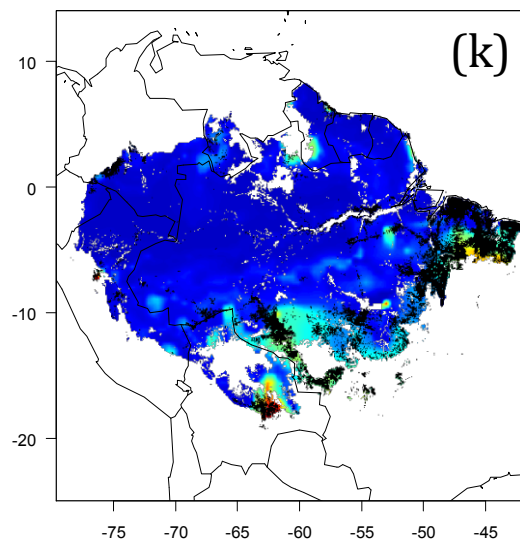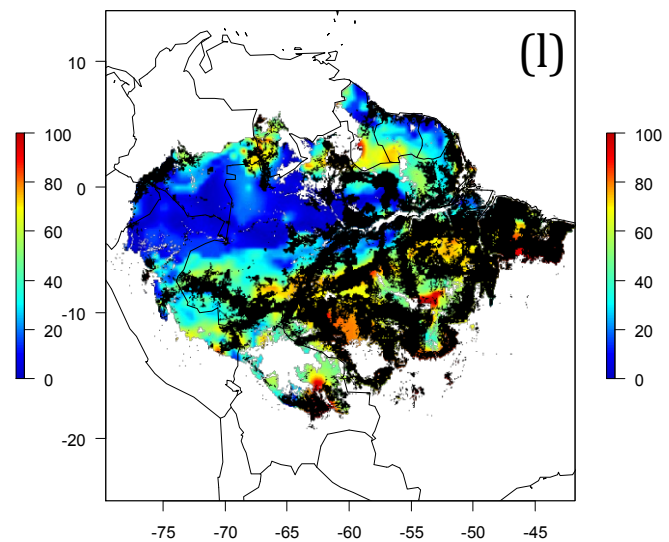

Supplement: Supplementary file 2 — Figure S1. The percent reduction in number of potential future climate analog source populations in Amazonia under 2070 climate projections accounting for climate analogs lost due to deforestation as of 2002 and under future BAU deforestation for 2050. Black represents a 100% reduction in available climate analogs based on losses from deforestation and the introduction of novel climates. [file ECE3-5-4315-s002.pdf]
